# Protein and synthetic polymer injection for induction of obstructive hydrocephalus in rats

**DOI:** 10.1186/1743-8454-4-9

**Published:** 2007-09-25

**Authors:** Ili Slobodian, Dmitri Krassioukov-Enns, Marc R Del Bigio

**Affiliations:** 1 Department of Pathology, University of Manitoba and Manitoba Institute of Child Health, Winnipeg, MB R3E 3P5, Canada

## Abstract

**Background:**

The objective of this study was to develop a simple and inexpensive animal model of induced obstructive hydrocephalus with minimal tissue inflammation, as an alternative to kaolin injection.

**Materials:**

Two-hundred and two male Sprague-Dawley rats aged 3 weeks received intracisternal injections of kaolin (25% suspension), Matrigel, type 1 collagen from rat tail, fibrin glue (Tisseel), n-butyl-cyanoacrylate (NBCA), or ethylene vinyl alcohol copolymer (Onyx-18 and Onyx-34). Magnetic resonance imaging was used to assess ventricle size. Animals were euthanized at 2, 5, 10 and 14 days post-injection for histological analysis.

**Results:**

Kaolin was associated with 10% mortality and successful induction of hydrocephalus in 97% of survivors (ventricle area proportion 0.168 ± 0.018). Rapidly hardening agents (fibrin glue, NBCA, vinyl polymer) had high mortality rates and low success rates in survivors. Only Matrigel had relatively low mortality (17%) and moderate success rate (20%). An inflammatory response with macrophages and some lymphocytes was associated with kaolin. There was negligible inflammation associated with Matrigel. A severe inflammatory response with giant cell formation was associated with ethylene vinyl alcohol copolymer.

**Conclusion:**

Kaolin predictably produces moderate to severe hydrocephalus with a mild chronic inflammatory reaction and fibrosis of the leptomeninges. Other synthetic polymers and biopolymers tested are unreliable and cause different types of inflammation.

## Background

Hydrocephalus is a common neurological condition characterized by enlargement of the cerebrospinal fluid (CSF)-filled ventricles, which leads to damage of surrounding brain tissue [1,2]. To study the pathogenesis of brain damage, hydrocephalus can be experimentally induced through a variety of techniques [3]. The most widely used method is through the injection of kaolin (aluminum silicate) into the cisterna magna [4]. This method is inexpensive, simple, reliable, minimally invasive leaving no visible wound, and to some extent titratable. Kaolin causes an inflammatory response of the leptomeninges and the subarachnoid space around the brainstem and cerebellum, thus causing obstruction at the fourth ventricle outlets [5,6]. In some respects the kaolin-induction method mimics hydrocephalus caused by meningitis. This model has been criticized for its inflammatory effects. Macrophages, CD4- and CD8- lymphocytes are evident in the subarachnoid space and, according to one report, in surrounding brain parenchyma [7], although this latter aspect has not been replicated in published literature or in our laboratory. Reactive microglia appear in the white matter surrounding the ventricles [8,9], but this is independent of the mode of induction. It is conceivable that the kaolin-induced inflammation modifies the brain damage, for example through release of cytokines, and for this reason additional options are desired.

Other materials that have been successfully utilized for hydrocephalus induction include bacteria, blood, cotton, lampblack, and silicone oil. Viscous silicone oil causes mild hydrocephalus associated with negligible inflammation, however, it is not a reliable method for small animal induction because it is difficult to force through a small-gauge needle [3,5]. Blood injection directly into the ventricles of young rats can cause hydrocephalus [10]. Unfortunately this directly disrupts brain tissue at the site of injection. We have attempted to induce hydrocephalus by injection of blood into the cisterna magna without success (Del Bigio; unpublished observations). Intracranial injection of growth factors such as fibroblast growth factor type 2 (FGF2) [11-13] and transforming growth factor beta (TGFbeta) [14] cause meningeal fibrosis and hydrocephalus in rodents, but both are extremely expensive and themselves can have direct effects on brain.

Our goal was to develop an inexpensive injection model of hydrocephalus in rats with minimal tissue inflammation. We evaluated a variety of protein and synthetic polymers 2–14d after injection. Among them were Matrigel^®^, collagen I from rat tail, fibrin glue (Tisseel^®^), n-butyl-cyanoacrylate (NBCA), and ethylene vinyl polymer (Onyx-18 and Onyx-34^®^). Kaolin was used as the control. Matrigel is a solubilized basement membrane preparation extracted from Engelbreth-Holm-Swarm (EHS) sarcoma cells [15], which at 37°C self-assembles into a gel [16]. We used the growth factor-reduced product, which is composed of laminin (61%), collagen IV (30%), and entactin (7%), with residual TGFbeta, FGF, and tissue plasminogen activator, as well as other growth factors. Collagen 1 is a major extracellular structural protein. As a gel, it can facilitate cell growth in the central nervous system [17]. Fibrin glue is a two-component surgical tissue adhesive used to control bleeding and block CSF leaks [18]. It is composed of fibrinogen and thrombin [19,20]. NBCA glue is a rapidly polymerizing agent that adheres to vessel walls and surrounding tissue; it is commonly used in endovascular surgical techniques [21]. It has been used to induce hydrocephalus in adult dogs [22]. Onyx^® ^is an ethylene vinyl alcohol copolymer dissolved in dimethyl sulfoxide (DMSO) and is combined with tantalum granules as a contrast agent. It is a non-adhesive embolic agent that develops into a non-permeable, flexible coagulate in the presence of an ionic environment, such as blood or CSF [23]. We hypothesized that at least one of these agents would be as efficacious as kaolin for induction of hydrocephalus in young rats.

## Methods

### Animal preparation

All animals were treated in accordance within the guidelines set by the Canadian Council on Animal Care. The local animal use committee approved the experimental procedures and all efforts were made to minimize the suffering and the number of animals used. Humane endpoints for euthanasia were established prior to experimentation to avoid unnecessary suffering, such as reduced mobility, respiratory distress and severe neurological impairment. At several points during the experiments we discussed modifications with the veterinarians. Agents or doses with obviously adverse effects were tested only once and then abandoned. Two hundred and two locally bred male Sprague-Dawley rats weighing 45–55 g at 3 weeks of age were used. Rats were anesthetized with isoflurane gas (1.5% in oxygen). The head and neck were shaved and the neck was flexed to maximize exposure to the foramen magnum. Under aseptic conditions, a 0.30 ml syringe with 30-gauge needle was used to inject each polymer into the cisterna magna percutaneously; 35 μl was the starting volume based on our previous experience with kaolin. Animals were monitored during recovery from anesthetic, weighed regularly, and observed for signs of neurological impairment. Rats were housed 4–5 to a cage and allowed food and water freely. An overdose of carbon dioxide gas was used to euthanize the animals.

### Polymer Preparation

Each polymer was tested in the same manner and all were sterile at the time of administration. The procedures were conducted in 4 batches of ~50 animals per batch. For the control group (10 in each batch), rats received injections of 30–35 μl sterile kaolin suspension (250 mg/mL in 0.9% saline; Sigma, St. Louis MO, USA). Fibrin glue was prepared according to the supplier (Tisseel^®^) (Baxter, Mississauga, Ontario, Canada) by mixing individual freeze-dried components in two separate syringes. The first component consisted of concentrated fibrinogen and aprotinin, and the second component consisted of thrombin and CaCl_2_. Using the Duplojet applicator supplied, 35–55 μl of mixed fibrin glue was injected into the cisterna magna. This procedure was abandoned after 2 batches. Liquid high concentration rat tail collagen I (BD Biosciences, Bedford, MA, USA) with a concentration range of 8–11 mg/mL was stored at 5°C until needed. Rats received 30–40 μl injections of undiluted collagen at room temperature. This procedure was abandoned after 2 batches. Undiluted NBCA (Vetbond, Sigma; St. Louis MO, USA) was injected at room temperature at volumes of 25–35 μl per animal. This procedure was also abandoned after 2 batches. Growth factor reduced Matrigel (BD Biosciences, Bedford, MA, USA) was stored at -20°C prior to use. This product gels at 10°C, therefore it was thawed to liquid consistency and was injected quickly before polymerization. This procedure was completed in 4 batches of animals with increasing volumes. Volume adjustments for Matrigel were made after the first batch showed partial success with an initial injection volume of 35 μl. The second batch received 75 μl, while the third and fourth groups received 125 μl. Onyx-18 (6% ethylene vinyl alcohol, 94% DMSO, viscosity 18 cps at 40°C) and Onyx 34 (8% EVOH, 92% DMSO, viscosity 34 cps) (EV3 International, Plymouth, MN, USA) injections were conducted in 4 batches. The suspensions were shaken vigorously for 15 minutes prior to use and the syringe was primed with DMSO prior to filling. Animals received 35, 25, or 15 μl injections. Note that this was the only non-aqueous agent injected; we did not include a DMSO only control group.

Animals from the first two batches were euthanized 14d after injection. Some animals from the 3^rd ^and 4^th ^batches were randomly selected for earlier euthanasia at 2, 5, and 10d to allow histological analysis of the early changes. The number of rats in each group and at each time point is shown in Table 1.

**Table 1:** Summary of polymer injection mortality and ventricular size index

Substance	Total Number Injected	Acute Mortality (a)	Time Points	Number with enlarged ventricles (b)	Ventricle Size Index (c)
Kaolin (30–35 μl)	40	4 (10%)	2d	4/4	
			5d	5/5	0.125 ± 0.024
			10d	4/4	
			14d	21/23	0.168 ± 0.018
Matrigel (d)	42	7 (17%)	2d	2/3	
		p = 0.520	5d	1/4	0.103 ± 0.020
			10d	1/1	
			14d	3/27	0.057 ± 0.021
				p < 0.001	p < 0.05
Onyx-18 (e)	29	13 (45%)	2d	1/3	
		p < 0.001	5d	0/3	
			10d	0/2	
			14d	0/8	-
				p < 0.001	
Onyx-34 (f)	37	10 (27%)	2d	2/6	
		p = 0.076	5d	3/5	0.113 ± 0.013
			10d	0/9	
			14d	2/7	0.135 ± 0.016
				p < 0.001	
NBCA (25–35 μl)	23	17 (74%)	5d	1/1	
		p < 0.001	14d	3/5	0.026 ± 0.016
				p = 0.091	p < 0.05
Tisseel (g)	19	11 (58%)	14d	0/8	-
		p < 0.001		p < 0.001	
Collagen type1 (30–40 μl)	12	0 (0%)	14d	0/12	-
		p = 0.562		p < 0.001	

a) Statistical comparison to kaolin using Fisher exact test (two-tailed)

b) Enlarged ventricles defined as ventricle area index >0.02. Statistical comparison to kaolin using Fisher exact test (two-tailed) based upon total survivors studied at all time points

c) Ventricle size index calculated on the MR slice immediately anterior the the third ventricle, total ventricle area/total brain area. The value shown is for successes only, mean ± SEM. Statistical comparison to kaolin based upon combined 10 day and 14 day data, ANOVA with Games-Howell post hoc intergroup analysis (which allows for different group sizes).

d) 35 μl 0/11 successes; 75 μl 2/10 successful; 125 μl 4/11 successful; 150 μl 10/10 fatal

e) 35 μl 7/7 fatal; 25 μl 1/7 successful; 15 μl 0/15 successful

f) 35 μl 8/8 fatal; 25 μl 5/9 successful; 15 μl 2/20 successful

g) 55 μl 9/9 fatal; 35 μl 2/10 fatal

### Magnetic resonance imaging

Magnetic resonance (MR) studies were performed prior to euthanasia using a Bruker Biospec/3 MR scanner equipped with a 21-cm bore magnet operating at a field of 7 Tesla (Karlsruhe, Germany) to obtain T2-weighted images of the brain in the coronal plane. The rats were anesthetized briefly with 1.5–2% isoflurane. These methods have been previously described in detail [24]. Ventricle sizes were blindly assessed on the image slice immediately anterior to the third ventricle by measuring the area of the ventricles and the area of the brain and calculating the ventricle to brain ratio. Successful induction of hydrocephalus in rats surviving >1d was defined qualitatively as ventricle size obviously greater than the largest control (ventricle area index >0.02).

### Histological analysis

After imaging, a carbon dioxide overdose was followed by perfusion with 10% formalin through cardiac puncture. The skin was removed and heads were further fixed with the brains in situ in 10% formalin. The heads were decalcified in 10% formic acid for 72 h followed by paraffin embedding. We chose to embed the entire head so that we could define the injected agents and resulting changes with respect to the brain and the surrounding meninges and skull. Samples including the cerebellum, cisterna magna and fourth ventricle were then cut into 6 μm sections, de-waxed and stained with hematoxylin and eosin, Masson's trichrome (to demonstrate collagen), and Leder stain (to demonstrate chloroacetate esterase activity in neutrophils). Blinded assessments were not possible because the administered agents were macroscopically and microscopically obvious.

### Statistics

Mortality rates and hydrocephalus induction success rates were compared to kaolin baseline using Fisher exact test. Ventricle size ratios among successes were compared using ANOVA with Games-Howell post hoc comparisons (which allows for groups of unequal size).

## Results

### Mortality

Rats tolerated the kaolin, Matrigel, and collagen injections fairly well with acceptable levels of acute mortality (10%, 17%, and 0% respectively died or were humanely euthanized within 24 h of injection) (see Table 1 for summary of results). Among the Matrigel subjects, rats with large volumes (125–150 μl) died immediately. The rapidly hardening agents were associated with significantly greater mortality. Fibrin glue produced 58% acute mortality (usually within minutes); this was due to rapid formation of a rubbery mass adjacent to the brainstem. NBCA injections seemed to be tolerated well initially with most rats awaking from the anesthetic. However, by 24 h 74% had died or were euthanized. We hypothesize that the cause of death of these particular animals was brain stem compression, as upon brain dissection we found hard glue deposits with sharp edges in the posterior fossa, which likely traumatized the brainstem, once the animals started moving. We saw no evidence of hemorrhage after removal of the brain in this region. There was a combined 36% mortality rate with the ethylene vinyl polymer groups either immediately or within 24 h after injection. The majority of deaths were among animals injected with 35 μl; mortality was lower with smaller volumes. Regardless of the volume, there was an unusual behavioral response to the ethylene vinyl polymer injections. Upon waking from anesthetic, rats scratched at their face and nose profusely and appeared hyperactive for a few minutes. This could be related to the DMSO solvent. With regard to the high mortality experienced by rats in some groups, we were able to abort planned injections of agents that caused immediate death, but the unanticipated early deterioration of NBCA injections was not detected until a second batch had received injections.

### Ventricular Dilatation

**Figure 1:**
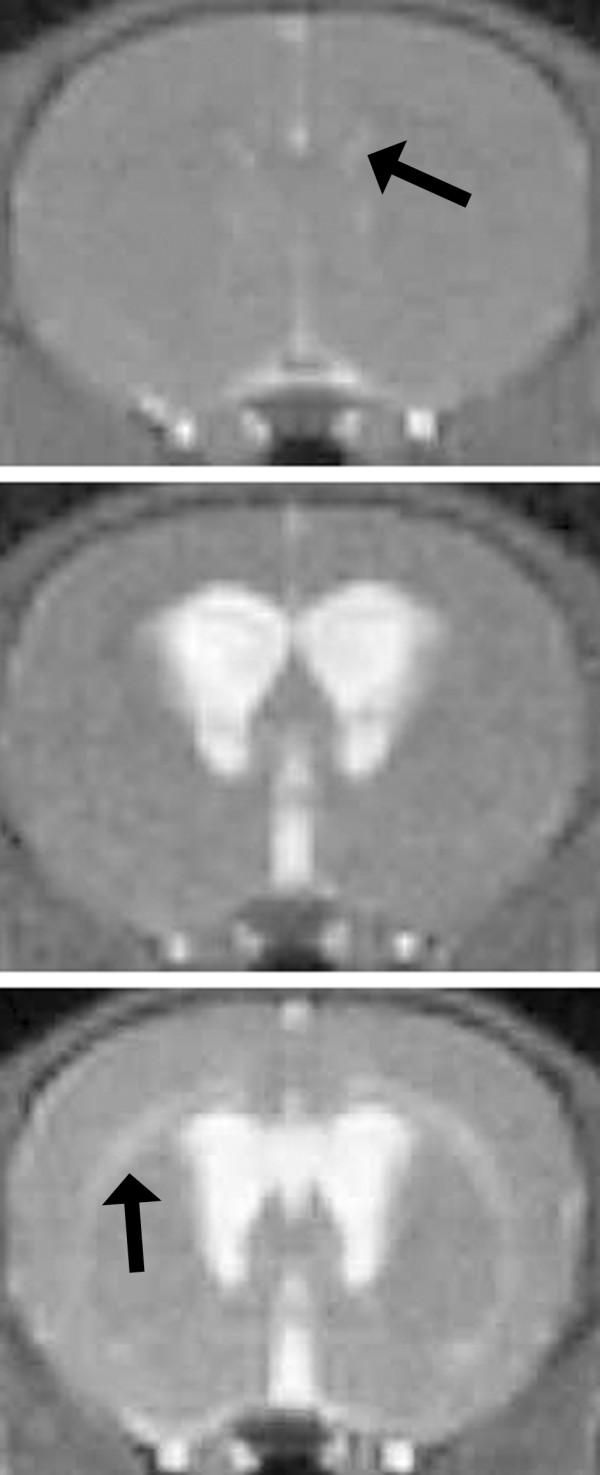
T2-weighted magnetic resonance images showing coronal slices through 5-week-old rat brain at the level of the anterior third ventricle. Cerebrospinal fluid (CSF) is bright. In normal rats (and induction failures) the ventricles are barely visible (upper panel; arrow). Kaolin injected at age 3 weeks is associated with marked enlargement of the lateral and third ventricles (middle panel; in this example the ventricle size index is 0.15). Other agents were less reliable. Matrigel caused moderate enlargement (lower panel; in this example the ventricle size index is 0.06). White matter edema is also apparent (arrow); it is usually associated with active enlargement.

Among those rats surviving ≥24 h, the ventricle size was determined using MR imaging (Figure 1). There were significant differences between the groups. Kaolin predictably produced a moderate to severe degree of hydrocephalus with a proportionate ventricle size of 0.168 ± 0.018 at 10–14d (see Table 1 for details). Matrigel was associated with a mild degree of hydrocephalus (ventricle size 0.057 ± 0.021). Among the few survivors, ethylene vinyl polymer was associated with ventriculomegaly similar to that from kaolin (0.135 ± 0.016).

### Histological results

**Figure 2:**
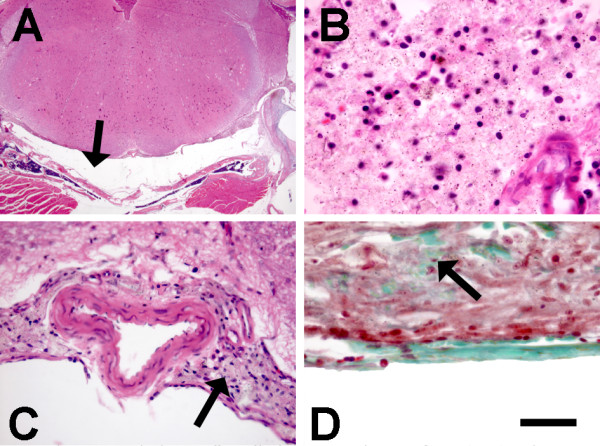
Photomicrographs showing effects of kaolin injection. The subarachnoid space ventral to the medulla is expanded (arrow in A; H&E stain) by aggregates of inflammatory cells and kaolin (dark granules) (B; H&E stain). Fourteen days post-injection (C; H&E stain), the kaolin has been engulfed by macrophages in the subarachnoid space (arrow); there is no abnormality apparent in the adjacent medulla. There is also arachnoid fibrosis (D; Masson's Trichrome stain) with green stained collagen (arrow) in the midst of the macrophages. Scale Bar (shown in frame D) is 50 μm for B and D, 100 μm for C, and 1600 μm for A.

There were major differences between groups with respect to the histological changes in the vicinity of the cisterna magna injection sites. Kaolin along the ventral surface of the brainstem was associated with mild accumulations of neutrophils, lymphocytes, monocytes and rare eosinophils within 2d of injection (Figure 2). By 5d, collagen deposition was apparent and kaolin had clearly been ingested by macrophages. There were minimal neutrophils and eosinophils by 10d. The brainstem and cerebellum adjacent to kaolin collections were histologically unaltered.

**Figure 3:**
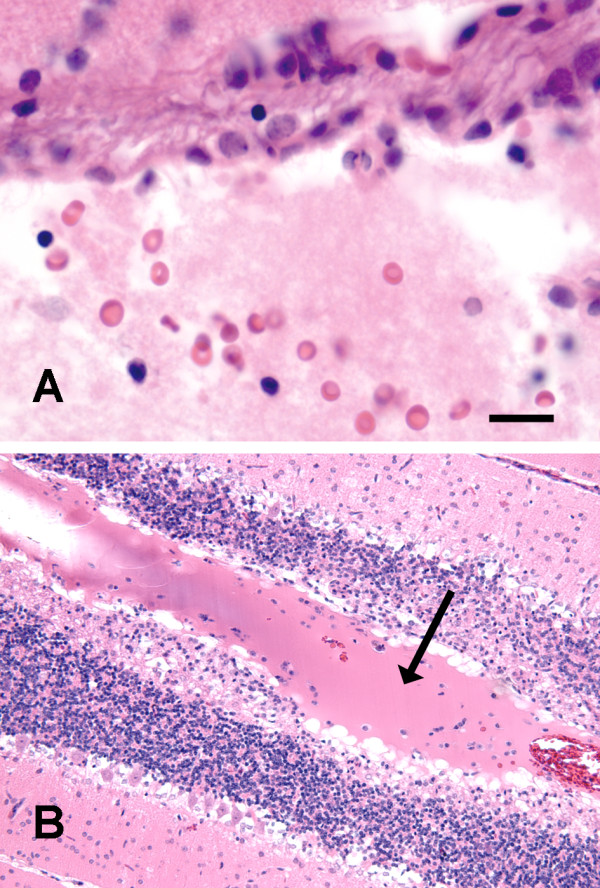
Photomicrographs showing effects of Matrigel injection. Two days post-injection (A; H&E stain) the ventral subarachnoid space is filled with Matrigel, which appears as an amorphous protein gel with scattered blood cells. Five to 14d post-injection (B; H&E stain), the Matrigel spreads around the cerebellum and into sulci (arrow) forming an acellular mass that does not elicit an inflammatory response but traps some of the blood cells. Scale Bar (shown in frame A) is 25 μm for A and 100 μm for B.

Matrigel injections were associated with neutrophils and lymphocytes, albeit to a lesser extent than kaolin. The Matrigel polymer appeared as an acellular homogenous faintly eosinophilic material in the subarachnoid space (Figure 3A). Matrigel spread well beyond the brainstem into some of the cerebellar sulci. It appeared to be surrounded by fibroblasts and hyperplastic small blood vessels with mild collagen accumulation at 5d. From 5–14d there was no evidence of vascular growth into the gel mass and there was no inflammation (Figure 3B). Collagen injections were characterized by similar changes at 14d (not shown).

**Figure 4:**
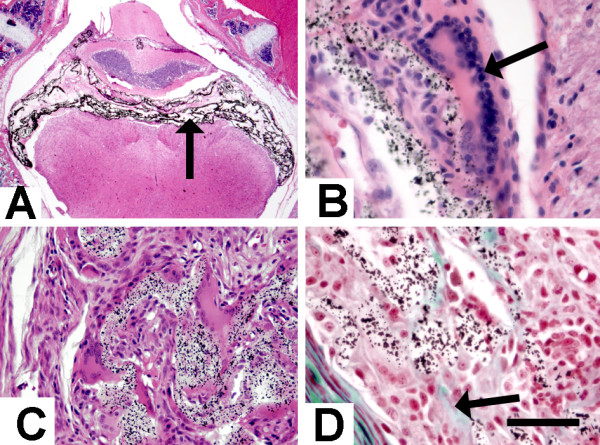
Photomicrographs showing effects of ethylene vinyl alcohol copolymer (Onyx) injection. At low magnification (A; H&E stain), the black tantalum granules in the polymer matrix are obvious in the fourth ventricle (arrow). By 2d (B; H&E stain) multinucleate (foreign body reaction) giant cells are apparent (arrow) adjacent to the Onyx. By 14d (C; H&E stain) the giant cell and fibroblastic reaction is more advanced, surrounding the Onyx clusters. There is associated collagen (green material, arrow) deposition as early as 5d (D; Masson's trichrome stain). Scale Bar (shown in frame D) is 50 μm for B and D, 100 μm for C, and 1600 μm for A.

Ethylene vinyl polymer appeared as a transparent material with black granules (tantalum) located in the fourth ventricle and lateral subarachnoid space (Figure 4A). Unlike kaolin and the proteins, it is viscous and does not spread into the ventral subarachnoid space. Arachnoid fibrosis and severe inflammatory responses (neutrophils and multinucleated foreign-body giant cells) were apparent at 2d post-injection (Figure 4B). At 5d there was an increase in the number of giant cells present, minimal neutrophils, and early collagen formation (Figure 4D). These changes persisted at 14d (Figure 4C).

Histological analysis of fibrin glue and NBCA was only done at 14d because it had become quickly apparent that there was no successful induction of hydrocephalus. Fibrin glue caused moderate arachnoid cell proliferation but only mild inflammation. NBCA caused moderate arachnoid cell proliferation without evidence of inflammation (not shown).

## Discussion

Kaolin injection is an effective method for producing hydrocephalus in almost all animal species tested [3]. Although this method offers dose-dependent responses to some extent, the response is variable. Furthermore, the inflammatory response, which consists largely of macrophages with some lymphocytes, that causes obstruction to CSF flow is a potentially confounding factor. Although it is an aluminum-based compound, in rats surviving 9 months after kaolin injection there is no evidence that the material spreads very far, nor is there an association in this model with formation of neurofibrillary tangles (Del Bigio; unpublished observations 2003). Injectable polymerizing agents, both protein based and synthetic, appeared to offer an effective alternative to kaolin because they solidify into a gel matrix that may conform to the subarachnoid compartment. The proteins Matrigel, collagen I, and fibrin have all been reported to support cell survival, growth and angiogenesis in cardiac muscle [25]. Injected into the cisterna magna, Matrigel spread locally in the subarachnoid compartment forming protein casts thereby retarding CSF flow and producing mild ventriculomegaly. Encouragingly, Matrigel associated-hydrocephalus caused no inflammation beyond that associated with the needle insertion. However, we could not attain the magnitude of ventriculomegaly possible with kaolin. Furthermore, in the course of the experiment we noticed differences between batches of Matrigel purchased from the supplier; one batch was less viscous than the first and larger volumes were required. One would need to address issues of initial protein concentration to avoid large volumes. It remains to be determined if the effect would be stable. It has been suggested that Matrigel, collagen I, and fibrin might be absorbed after ~5 weeks [25]. The age of the rats used should be considered. In our experience with rats ranging from newborn to adult there is little variation with respect to the response to kaolin, although other species such as sheep exhibit more lymphocytic response to kaolin (unpublished data). We expect that adult rats would respond in a similar manner and that younger rats might suffer more serious complications with the rapidly hardening agents because their fourth ventricle and cisterna magna are smaller.

We expected collagen to form a protein plug with minimal inflammation [17]. However, it was entirely ineffective. In subsequent in vitro experiments we found that the collagen gel dispersed rapidly in artificial CSF (not shown). Fibrin glue is a widely used surgical tissue adhesive [18] and is stable in CSF [26]. In experimental application to brain surface it causes an inflammatory reaction that peaks at ~1 week and persists with lymphocytes and foreign body giant cells for weeks [27,28]. We encountered numerous problems with the administration of the fibrin glue. Because it is highly viscous, we found it extremely difficult to apply through a fine needle. The compounds would solidify immediately on contact with each other and therefore clog the applicator or form a bulky mass in the cisterna magna that compressed the brainstem.

The two synthetic agents tested, N-butyl-cyanoacrylate (NBCA) and ethylene vinyl alcohol copolymer (Onyx), were unacceptable for different reasons. NBCA is a rapidly polymerizing agent that adheres to vessel walls and surrounding tissue [21]. The chronic local tissue irritation and inflammation associated with cyanoacrylates is related to the degradation products of the polymer, which include formaldehyde and alkyl cyanoacetate [29,30]. Although NBCA has been useful for production of hydrocephalus in dogs, the jagged forms adjacent to the brainstem proved fatal for largely mechanical reasons. Polyvinyl alcohols are also used for endovascular embolism treatments. Unlike NBCA, they are non-adhesive and flexible, and produce less inflammation [30]. We observed intense foreign body giant cell type reaction in response to Onyx, consistent with the reported vascular responses [31-33]. It has been suggested that DMSO can cause severe inflammation following intra-arterial injection [23], however, DMSO has also been used as an anti-inflammatory agent [34].

## Conclusion

Based on our observations, we conclude that none of the agents tested, which included many of the currently available substances used for intracranial applications in humans, are better than kaolin for use in rats. Rapidly polymerizing agents form solid masses that can damage the brainstem of rodents. Pliable synthetic polymers are associated with intense foreign-body type inflammatory reactions. The protein mixture Matrigel might be useful for inducing mild hydrocephalus, because it is easy to work with and causes minimal inflammation. However, we found inconsistencies between batches, and the longevity of the obstruction is unknown. Further study of this protein polymer is warranted. However, until efficacy can be shown, we plan to continue use of kaolin, which reliably and inexpensively produces severe ventriculomegaly in experimental animals. The caveat is that one must be willing to accept the remote possibility that the inflammation is a confounding factor in hydrocephalus-induced brain damage.

## Competing interests

The author(s) declare that they have no competing interests.

## Authors' contributions

IS made substantial contributions to conception, design, analysis and interpretation of data, and drafted the manuscript. She performed all animal injections, monitoring and euthanasia. DK-E contributed to design, animal monitoring, euthanasia, and manuscript development. MRDB conceived the study and participated in its design and coordination, finalized the manuscript, and performed the statistical analyses. All authors have read and approved the final version of the manuscript.
